# Sleep on it!

**DOI:** 10.1093/braincomms/fcaf072

**Published:** 2025-03-04

**Authors:** Laurent Sheybani

**Affiliations:** London, UK

## Abstract

Our Associate Editor, Laurent Sheybani, discusses some very old and very recent findings on sleep physiology and function, hoping to raise further interest and publications in the field.

## Graphical Abstract

**Graphical Abstract:**
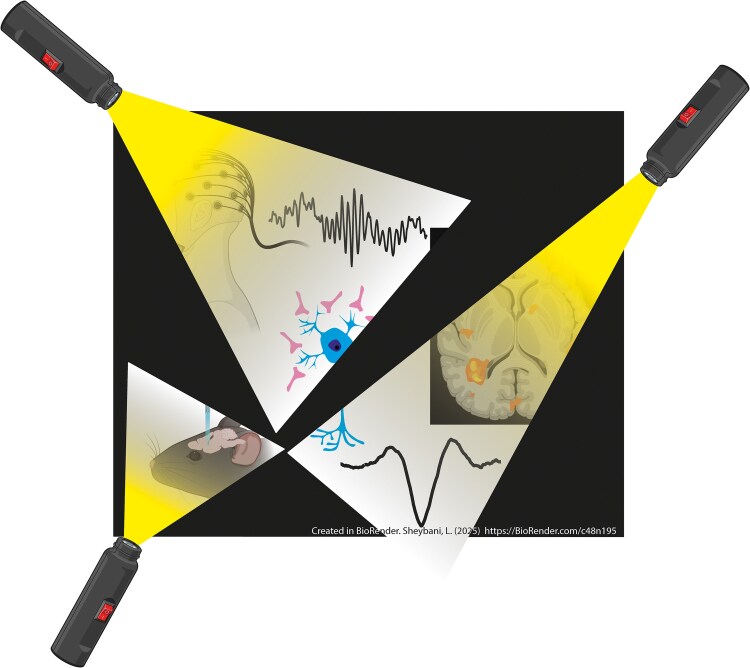

In Greek mythology, *Hypnos* (Υπνος, Somnus in Latin, from which the word ‘somnology’, the science of sleep, originates) is the son of *Nyx* (Νύξ, the night) and the twin brother of *Thanatos* (Θανατος, death). Although Nyx can be translated as ‘night’, it also refers to obscurity and darkness.^1^ Thus, what we see today as a state of recovery and refreshment was, quite the opposite, associated with an absence of life. In his thesis on sleep, Aristotle used the word *pathos* (πάθος) to refer to sleep.^2^ This does not translate into ‘disease’, as one might expect (the word pathology comes from *pathos*). Rather, *pathos* refers to a condition that is imposed on someone, as opposed to a condition that emerges or starts from someone, or is generated by someone.^1^ While it is true that environmental factors influence sleep,^3-5^ the idea that sleep is imposed upon us, rather than being an internally generated state, is now obsolete. Thus, not only was sleep seen as a dark, lifeless condition, but also one that derived from external influence.

Sleep is thus a mysterious state, surrounded by legends since the dawn of time. Some of them have a kernel of truth, including the locution ‘Sleep on it!’, which alludes to its restorative function. Indeed, the restorative function of sleep is an active field of research that has generated numerous publications in recent years. One way to assess this restorative function of sleep is to measure the flow of the CSF within the glymphatic system—a perivascular network involved in waste clearance—across sleep and wakefulness. This was tested in a study published in 2013.^6^ In their work, Xie *et al.*^6^ injected a tracer in the cisterna magna of mice, a brain compartment filled with CSF, to examine the movement of that tracer across vigilance states. They observed that the flux of the tracer was enhanced during sleep, which suggested increased function of the glymphatic system during sleep. A few years later, increased pulsation of the CSF was identified during sleep, in comparison with wake, in humans.^7^ This CSF drainage has been recently shown to be driven by pulsations of norepinephrine,^8^ which also controls cycles across the different sleep stages.^9^ Interestingly, norepinephrine pulsations can also foster microarousals during sleep.^9^ Hence, although sleep interruptions—by microarousals—were believed to be detrimental to sleep quality—and they are probably to a certain extent—they could also subserve some physiological functions.^10^

In line with the increased drainage of CSF during sleep,^6,7^ increased level of β-amyloid protein—a key component of the pathogenesis of Alzheimer’s disease—was observed in the CSF of sleep-deprived human subjects.^11^ However, in contrast with these findings, a recent study published in *Nature Neuroscience* provided evidence that brain clearance is rather reduced during sleep.^12^ To explain the discrepancy, Miao *et al.*^12^ (i.e. the authors of this study) highlighted the fact that enhanced clearance during sleep in Xie *et al.* was assessed by high levels of a dye in the cortex, after injection in the cisterna magna, but that concentration could have been high either because of increased convective force from the cisterna magna into the cortex (high clearance) or because of decrease convective force outside the brain (low clearance). In contrast, Miao *et al.* injected a dye in the caudate putamen and then measured its level in the frontal cortex. Thus, different methodological approaches might have contributed to the opposing results. However, this does not explain why Xie *et al.*^6^ also found increased clearance of a tracer (^14^C-inulin, an inert molecule) and, again, of β-amyloid. Indeed, in the latter experiment, both the tracer and β-amyloid were injected in the cortex, not the cisterna magna, making the remaining level of these compounds a function of their rate of clearance only. While more recent results support increased CSF drainage during sleep,^8^ it thus remains to be clarified why such different results were observed in these two studies.

Hence, some evidence for a restorative function of sleep can be observed at the molecular level. Elsewhere, evidence suggests that it can also be observed at the level of neuronal activity. The concept of homeostatic sleep regulation suggests that brain activity is reset during sleep to compensate for enhanced activity during the previous wake period. For example, the recruitment of a specific brain region during wake is associated with increased sleep-related activity during the subsequent sleep period.^13^ More generally, while some neurons increase and others decrease firing during sleep, the net effect of sleep is a decrease of activity.^14^ Thus, quantitative analyses of neuronal activity indeed support the locution that we should ‘sleep on it’.

Could ‘sleep on it’ also suggest that sleep helps you carry out particular tasks? In French, we say ‘la nuit porte conseil’: ‘sleep provides the necessary advice’. A famous example of this is the discovery of the benzene molecule (a ring of 6 carbon atoms) by Auguste Kekulé. He reported seeing a snake biting its own tail in a dream, which led him to picture a circular configuration of benzene.^15^ Do we have any scientific evidence for the snake-biting-tail phenomenon? Recently, a team of French scientists found that spending a few seconds in light sleep improved the ability to solve a mathematical problem.^16^ Interestingly, if people slept further into a deeper stage of sleep, the beneficial effect of sleep disappeared. Sleep on it—but not too much.

In the same line, we have probably all been told that listening to items to be remembered during sleep improves memory. Who has never dreamt to learn their German/Spanish/Italian/French/English/Chinese vocabulary only by listening, during sleep, to a pre-registered version of it? (Did it work?) Recent studies have shown that there might be some level of *implicit* memory encoding during sleep,^17,18^ such as electroencephalographic (EEG) or behavioural signature of encoding without explicitly remembering the new items. In contrast, there is no strong evidence of *explicit* memory encoding during sleep.^17,19^ For example, when people are presented with a list of words during sleep, they fail to reliably recognize them the next morning.^17^ However, EEG activity at word presentation exhibits slight differences between words that were presented during sleep and those presented for the first time during the next wake period, indicating some signature of *implicit* memory during sleep.^17^ Contrasting with the potential *implicit* encoding function of sleep, one study showed that presenting new information during sleep could prevent its subsequent learning during wake. In their study, Andrillon *et al.*^20^ presented different types of sounds to people while sleeping. During the subsequent wake periods, they observed decreased performance in learning to recognize those sounds that were previously presented during sleep. Hence, in contrast to the well-known myth that we can learn during sleep, this study rather suggests an impeding role of sleep.

Sleep is a fascinating topic, owing both to the exciting scientific questions it raises and the historical perspective it provides. I hope this editorial will raise further interest in this field and I invite you to submit your exciting research on sleep, sleep disorders and the interactions between sleep physiology and neurological—or not neurological—conditions to *Brain Communications.*

The cover for this issue comes from Pini *et al*.^21^ and illustrates the brain connectome, which is altered in neurological disorders such as neurodegenerative diseases, representing potential targets for novel therapeutic approaches.
